# Genetic Relationship between Schizophrenia and Nicotine Dependence

**DOI:** 10.1038/srep25671

**Published:** 2016-05-10

**Authors:** Jingchun Chen, Silviu-Alin Bacanu, Hui Yu, Zhongming Zhao, Peilin Jia, Kenneth S. Kendler, Henry R. Kranzler, Joel Gelernter, Lindsay Farrer, Camelia Minica, Rene Pool, Yuri Milaneschi, Dorret I. Boomsma, Brenda W. J. H. Penninx, Rachel F. Tyndale, Jennifer J. Ware, Jacqueline M. Vink, Jaakko Kaprio, Marcus Munafò, Xiangning Chen, Cotinine meta-analysis group, Jennifer J. Ware, Xiangning Chen, Jacqueline M. Vink, Anu Loukola, Camelia Minica, Rene Pool, Yuri Milaneschi, Massimo Mangino, Cristina Menni, Jingchun Chen, Roseann Peterson, Kirsi Auro, Leo-Pekka Lyytikäinen, Juho Wedenoja, Alex I. Stiby, Gibran Hemani, Gonneke Willemsen, Jouke Jan Hottenga, Tellervo Korhonen, Markku Heliövaara, Markus Perola, Richard Rose, Lavinia Paternoster, Nic Timpson, Catherine A. Wassenaar, Andy Z. X. Zhu, George Davey Smith, Olli Raitakari, Terho Lehtimäki, Mika Kähönen, Seppo Koskinen, Timothy Spector, Brenda W. J. H. Penninx, Veikko Salomaa, Dorret I. Boomsma, Rachel F. Tyndale, Jaakko Kaprio, Marcus Munafò, FTND meta-analysis group, Jennifer J. Ware, Xiangning Chen, Jacqueline M. Vink, Anu Loukola, Camelia Minica, Jingchun Chen, Roseann Peterson, Nic Timpson, Michelle Taylor, Dorret I. Boomsma, Jaakko Kaprio, Marcus Munafò, Hermine Maes, Brien Riley, Kenneth S. Kendler, Joel Gelernter, Richard Sherva, Lindsay Farrer, Henry R. Kranzler, Brion Maher, Michael Vanyukov

**Affiliations:** 1 Nevada Institute of Personalized Medicine, University of Nevada at Las Vegas, 4505 S. Maryland Parkway, Las Vegas, NV 89154, USA; 2 Virginia Institute for Psychiatric and Behavioral Genetics, Virginia Commonwealth University, 800 E. Leigh Street, Richmond, VA 23298, USA; 3 Department of Psychiatry Virginia, Commonwealth University, 800 E. Leigh Street, Richmond, VA 23298, USA; 4 Departments of Biomedical Informatics and Psychiatry, Vanderbilt University School of Medicine, Nashville, TN 37203, USA; 5 Department of Psychiatry, University of Pennsylvania Perelman School of Medicine and VISN4 MIRECC, Philadelphia VA Medical Center, Philadelphia, PA, USA; 6 Division of Human Genetics, Department of Psychiatry, Yale University School of Medicine, and VA CT Healthcare Center, New Haven, CT 06516, USA; 7 Department of Medicine (Biomedical Genetics), Boston University School of Medicine, Boston, MA, USA; 8 Department of Biological Psychology, VU University, Amsterdam, Netherlands; 9 Department of Psychiatry and EMGO Institute for Health and Care Research, VU University Medical Center, Amsterdam, Netherlands; 10 Department of Pharmacology and Toxicology, University of Toronto, Toronto, Canada; 11 MRC Integrative Epidemiology Unit, University of Bristol, Bristol, UK; 12 Department of Biological Psychology, Vrije Universiteit, Amsterdam, The Netherlands; 13 Behavioural Science Institute, Radboud University, Nijmegen, The Netherlands; 14 University of Helsinki, Department of Public Health, P.O.Box 41 (Mannerheimintie 172), 00014 Helsinki, Finland; 15 National Institute for Health and Welfare, Department of Mental Health and Substance Abuse Services, P.O. Box 30 (Mannerheimintie 166), 00300 Helsinki, Finland; 16 University of Helsinki, Institute for Molecular Medicine, P.O. Box 20 (Tukholmankatu 8), 00014 Helsinki, Finland; 17 UK Centre for Tobacco and Alcohol Studies, School of Experimental Psychology, University of Bristol, Bristol, UK; 18 Department of Psychology, University of Nevada, Las Vegas, 4505 S. Maryland Parkway, Las Vegas, NV 89154, USA.; 19 Department of Twin Research and Genetic Epidemiology, King’s College London, London, United Kingdom.; 20 National Institute for Health and Welfare, Helsinki, Finland.; 21 Department of Clinical Chemistry, Fimlab Laboratories, Finland.; 22 School of Social and Community Medicine, University of Bristol, Bristol, United Kingdom.; 23 Department of Psychological and Brain Sciences, Indiana University, Indiana, United States.; 24 Department of Clinical Physiology and Nuclear Medicine, Turku University Hospital, Finland.; 25 Department of Clinical Physiology, Tampere University Hospital, Finland.; 26 Department of Mental Health, Johns Hopkins University, Baltimore, MD, USA.; 27 Department of Pharmaceutical Sciences, University of Pittsburgh, Pittsburgh, PA, USA.

## Abstract

It is well known that most schizophrenia patients smoke cigarettes. There are different hypotheses postulating the underlying mechanisms of this comorbidity. We used summary statistics from large meta-analyses of plasma cotinine concentration (COT), Fagerström test for nicotine dependence (FTND) and schizophrenia to examine the genetic relationship between these traits. We found that schizophrenia risk scores calculated at P-value thresholds of 5 × 10^−3^ and larger predicted FTND and cigarettes smoked per day (CPD), suggesting that genes most significantly associated with schizophrenia were not associated with FTND/CPD, consistent with the self-medication hypothesis. The COT risk scores predicted schizophrenia diagnosis at P-values of 5 × 10^−3^ and smaller, implying that genes most significantly associated with COT were associated with schizophrenia. These results implicated that schizophrenia and FTND/CPD/COT shared some genetic liability. Based on this shared liability, we identified multiple long non-coding RNAs and RNA binding protein genes (DA376252, BX089737, LOC101927273, LINC01029, LOC101928622, HY157071, DA902558, *RBFOX1* and *TINCR*), protein modification genes (*MANBA*, *UBE2D3*, and *RANGAP1*) and energy production genes (*XYLB*, *MTRF1* and *ENOX1*) that were associated with both conditions. Further analyses revealed that these shared genes were enriched in calcium signaling, long-term potentiation and neuroactive ligand-receptor interaction pathways that played a critical role in cognitive functions and neuronal plasticity.

Large scale genome wide association studies (GWASs) have identified risk genes for many complex human diseases and traits (http://www.genome.gov/gwastudies/), including psychiatric disorders such as schizophrenia and nicotine dependence (ND)^1^,^2^,^3^. These GWASs also show that many human diseases and traits are polygenic in nature and the contribution of individual genes is limited^4^,^5^,^6^. Many of these studies have been deposited in the database of Genotypes and Phenotypes (dbGaP, http://www.ncbi.nlm.nih.gov/gap) and are available for secondary analyses. These datasets provide an opportunity to examine the genetic relationship between correlated traits, and to discover and identify risk genes shared across these traits.

Pleiotropy is a phenomenon in which a single locus affects multiple traits^7^,^8^. It accounts for at least a part of the genetic mechanism of many correlated human behaviors and diseases. Pleiotropy can take two forms: either a single process, leading to a cascade of downstream effects (sometimes described as “mediated pleiotropy”), or a single locus influencing multiple traits (sometimes described as “biological pleiotropy”)^9^. Schizophrenia is highly comorbid with cigarette smoking^10^. However, the underlying biology of this comorbidity is not well understood^11^. Several hypotheses have been proposed. The self-medication hypothesis postulates that schizophrenia patients smoke to reduce symptoms and antipsychotics-induced side effects and to improve their attention and working memory^12^. Alternatively, schizophrenia and ND could have shared some genetic liability (i.e., biological pleiotropy)^13^, which is supported by recent studies of individual genes^14^,^15^,^16^,^17^,^18^,^19^,^20^,^21^. A third possibility is that smoking may be causal to schizophrenia (i.e., mediated pleiotropy)^22^. To explore the genetic relationship between schizophrenia and ND, we obtained the GWAS summary statistics from the Psychiatric Genomics Consortium (PGC) schizophrenia analyses and ND related traits from our unpublished studies, and conducted polygenic analyses. Under the hypothesis of biological pleiotropy, we expect that genetic risk scores of schizophrenia and ND related traits predict each other; whereas self-medication would anticipate unidirectional (schizophrenia to ND traits) prediction. In this article, we report the findings from these analyses.

## Results

### Nicotine dependence and schizophrenia share genetic liability

In these analyses, we calculated genetic risk scores for schizophrenia (supplementary Figure 1A) and tested whether the risk scores predicted FTND and CPD. The results were summarized in Table 1. Schizophrenia risk scores predicted FTND score and CPD at the thresholds of P ≤ 5 × 10^−3^, 5 × 10^−2^ and 5 × 10^−1^. The correlation coefficients at these thresholds were all positive, suggesting that a schizophrenia diagnosis was positively associated with cigarette smoking, consistent with the well-known comorbidity between schizophrenia and ND. However, schizophrenia risk scores explained only a very small fraction of the FTND and CPD traits.

FTND and COT risk scores (supplementary Figure 1B,C) were calculated for the subjects of the phase I PGC schizophrenia GWAS samples^23^ using the summary statistics from the FTND (n = 17,781) and COT (n = 4,548) GWAS meta-analyses. We then evaluated whether the genetic risk scores of COT and FTND could predict the schizophrenia diagnosis using logistic regression. The results were presented in Table 2. The COT risk scores calculated at the P-values of 5 × 10^−5^, 5 × 10^−4^ and 5 × 10^−3^ predicted schizophrenia diagnosis, but FTND risk scores failed to do so. For the P-values thresholds at which the COT risk scores predicted schizophrenia diagnosis, the beta coefficients were also positive, again, confirming the positive phenotypic correlation between ND and schizophrenia.

### Identification of shared variants between ND and schizophrenia

Our reciprocal polygenic analyses suggested that there were some shared genetic liability between schizophrenia and ND as defined by the FTND and COT traits. We then proceeded to identify the variants associated with both schizophrenia and ND traits. We computed joint P-values for each marker using the summary statistics from the schizophrenia and COT/FTND/TFC meta-analyses, and assigned a q-value to each of the joint P-values using an FDR method^24^,^25^. Table 3 listed the loci identified by the joint analyses with q-values ≤ 0.05. From the joint analyses between schizophrenia and COT, 11 loci reached genome-wide significance for association with both COT and schizophrenia, of which 2 loci had no known genes nearby and 6 were spliced ESTs or long non-coding RNAs. In the analyses between schizophrenia and FTND, 10 loci were identified, and 3 of them were ESTs or non-coding RNAs. The joint analyses between schizophrenia and TFC yielded 15 significant loci. The *CHRNA5-CHRNA3-CHRNB4* locus was the only one identified by all three smoking traits. In addition to some genes known to be associated with schizophrenia (HLA-B and MAD1L1), we also identified novel non-coding RNAs and RNA binding protein genes (DA376252, BX089737, LOC101927273, LINC01029, LOC101928622, HY157071, DA902558, *RBFOX1* and *TINCR*), post-translation modification genes (*MANBA*, *UBE2D3* and *RANGAP1*) and energy production genes (*XYLB*, *MTRF1* and *ENOX1*).

### Pathway enrichment and network interaction analyses

We further explored the pathways shared by schizophrenia and ND by selecting all markers with q-values less than 0.16 from the joint analyses between schizophrenia and smoking traits. After mapping the markers to genes, the genes showing potential association with schizophrenia and COT/FTND/TFC were pooled to search for pathways enriched in both conditions. In these analyses, we selected only the genes identified by at least 2 of the 3 smoking traits, yielding a total of 146 genes. After filtering out the human leukocyte antigen genes (*HLA-B*, *HLA-C*, *HLA-DOA*, *HLA-DQA1*, *HLA-DQB1*, *HLA-DRB1*, and *HLA-G*) due to their strong linkage disequilibrium^26^, we used the remaining 139 genes in pathway analyses.

**Figure 1:**
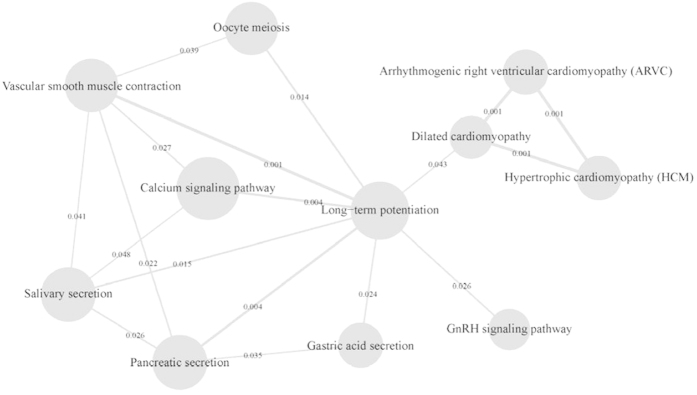
Pathway crosstalk network. The size of the node is proportional to the P-values of pathway enrichment test. The thickness of the edge is proportional to the P-values of pathway crosstalk.

Our analyses identified 16 unique pathways that were shared between schizophrenia and ND (Table 4). The most noticeable pathways were Calcium Signaling, Long-Term Potentiation, Neuroactive Ligand-Receptor Interaction, Phosphatidylinositol Signaling, Cell Adhesion Molecules, and Regulation of Actin Cytoskeleton pathways. Some of these pathways (Calcium Signaling, Long-Term Potentiation, Cell Adhesion Molecules, and Regulation of Actin Cytoskeleton) had been reported to be involved in schizophrenia^27^,^28^,^29^,^30^,^31^,^32^, others (Cell Adhesion Molecules and Neuroactive Ligand-Receptor Interaction) had been implicated in ND^33^,^34^. We found that these pathways were enriched in the genes associated with both ND and schizophrenia. Additionally, pathways involved in cardiomyopathy, GnRH signaling, gastric acid secretion and Alzheimer’s disease were also found to be shared between schizophrenia and ND. In the pathway network interaction analyses, we found a network of crosstalk between pathways (Fig. 1), with the Long-Term Potentiation located at the center of these interactions.

## Discussion

It is well known in psychiatric clinics that a large proportion of schizophrenia patients smoke cigarettes and smoke heavily^13^. The dominant hypothesis to explain the comorbidity is self-medication^12^, i.e., that schizophrenia patients smoke to ameliorate impairments in cognitive function and suppress psychotic symptoms. Another hypothesis contends that schizophrenia and ND share some genetic liability, and the high prevalence rate of cigarette smoking in schizophrenia patients is a manifestation that is partially due to the common liability^13^. A third possibility is that smoking may be a risk factor for the development of schizophrenia, given that smoking initiation typically predates the onset of schizophrenia^22^. These three hypotheses are not mutually exclusive, and all three may contribute to the observed co-occurrence of schizophrenia and smoking.

Previous studies examining this issue have largely focused on individual functions/symptoms or genes using relatively small sample sizes. Here we took a systematic approach, and examined the entire genome using large GWAS datasets and multiple traits. We observed different patterns between the reciprocal polygenic analyses (comparing Tables 1 and 2). When we used the genetic risk scores of schizophrenia to predict ND traits, the association was evident at P-values ≥ 5 × 10^−3^, with the association strength increased as the P-value threshold became larger (Table 1). Given that the PGC schizophrenia GWAS did not control for smoking status and quantity, and there was a large difference of smoking prevalence between schizophrenia patients and controls (on average, 65% or more schizophrenia patients smoke, and about 20% people smoke in the general population), we would expect that the PGC schizophrenia GWAS identify top candidates for ND related traits. But what we found was not the case. These top ranked candidates (i.e. those with P-values ≤ 5 × 10^−5^) from the PGC schizophrenia meta-analysis^1^ were not predict ND related traits. A likely explanation for these results is that genes most strongly associated with schizophrenia do not directly contribute to the smoking behaviors in schizophrenia patients. In other words, the reason why schizophrenia patients smoke is that they want to improve their cognitive functions and to suppress psychotic symptoms, not because that they are addicted to nicotine as regular smokers in the general population do. These results are consistent with the self-medication hypothesis.

In contrast, when we used COT risk scores to predict schizophrenia diagnosis, we found that smaller P-values produced stronger signals (Table 2), indicating that genes most strongly associated with ND were associated with schizophrenia. The results imply that either ND and schizophrenia share some genetic liability, or ND is a risk factor of schizophrenia. These fit the predictions of the shared liability hypothesis and that smoking is a causal risk for schizophrenia. Of note, these two explanations are not mutually exclusive. But without data on smoking of the patients we are unable to test the latter possibility (e.g., by stratifying our sample on smoking status).

Assuming biological pleiotropy to be the underlying mechanism, we devised a test to discover the variants shared between ND and schizophrenia. Using this approach, we identified multiple genes associated with both conditions (Table 3). Of these genes, the *CHRNA5*-*CHRNA3*-*CHRNB4* cluster had been found to be associated with CPD^2^,^3^ and other ND related traits, and it was reported to be associated with schizophrenia in the latest schizophrenia GWAS meta-analysis from PGC^1^. Several of the genes had been reported to be associated with schizophrenia (*HLA-B* and *MAD1L1*)^28^ and epilepsy (*KCNT1*, *PRICKLE2* and *RBFOX1*)^35^,^36^,^37^, suggesting that they might play a role in smoking behaviors as well. Our analyses also identified some novel genes shared between schizophrenia and ND, including a group of long non-coding RNAs and RNA binding protein genes (DA376252, BX089737, LOC101927273, LINC01029, LOC101928622, HY157071, DA902558, *RBFOX1* and *TINCR*), a group of post-translation modification genes (*MANBA*, *UBE2D3*, and *RANGAP1*) and a group of energy production genes (*XYLB*, *MTRF1* and *ENOX1*). Long non-coding RNAs were suggested to play a role in schizophrenia^38^,^39^, the identification of multiple long non-coding RNAs was intriguing.

Phenotype comorbidity is common in complex diseases and traits^7^,^8^. Pleiotropy, or shared genetic liability, may be an underlying mechanism of these comorbidities. Under this condition, different approaches have been developed to identify genes shared by the comorbid conditions^40^,^41^, and these approaches seem more powerful than standard GWAS^8^,^42^. Another advantage of these methods is that they can use the large number of GWAS datasets produced by a single phenotype/trait analyses. The approach we used to identify these shared loci is conservative. In our analyses, we excluded all markers reaching genome-wide significance from both schizophrenia and smoking traits and required a balanced contribution from both traits. Under this condition, if a marker reached genome wide significance for schizophrenia but had a modest association with ND traits (say P-values between 10^−4^ to 5 × 10^−6^), it was excluded from our joint testing. Similarly, some markers would be excluded if they reached genome wide significance in ND traits. Because the GWASs used have different sample sizes, and therefore varied in their statistical power, it is inevitable that we would miss some markers from the more powerful GWAS when we required balanced summary statistics in the joint testing.

Our pathway analyses identified multiple pathways shared by schizophrenia and ND. The most significant pathways were Calcium Signaling, Long-Term Potentiation and Neuroactive Ligand-Receptor Interaction. These pathways are involved in neurotransmitter transduction and communication between neurons, and they are essential for cognitive functions. These pathways have been shown to be involved in schizophrenia^28^,^43^,^44^ and ND^45^,^46^. The Cell Adhesion Molecules and Regulation of Actin Cytoskeleton pathways have also been reported in schizophrenia^31^,^47^,^48^,^49^ and ND^45^,^50^,^51^. Thus, our results are consistent with these studies. It is worth noting that the cardiomyopathy pathways were identified in our analyses and that, in a previous study, we found that *CMYA5* was associated with schizophrenia^52^. Another gene, *NDUFV2*, causative to hypertrophic cardiomyopathy^53^,^54^, the genetic form of cardiomyopathy, was also found to be associated with schizophrenia^55^,^56^,^57^. Pathway crosstalk analyses showed that many of these pathways interact with each other and together they form an interlinked network with the Long-Term Potentiation pathway at the center of these interactions. In animal studies, nicotine alters long-term potentiation^58^,^59^,^60^ and learning and memory^61^. In humans, smoking may alleviate cognitive impairment^62^, and both nicotine withdrawal and schizophrenia are associated with cognitive impairments^63^,^64^. Thus, compensating cognitive impairments may be a common motivational factor between regular smokers and schizophrenia patients.

In summary, our results supported the self-medication hypothesis. We also found evidence that schizophrenia and ND share some genetic liability and these results did not contradict the hypothesis that smoking was a causal risk factor for schizophrenia. Assuming shared liability and a balanced contribution, we identified novel candidate genes associated with both schizophrenia and ND. Analyses of the shared genes revealed multiple pathways and an interacting network centered on long-term potentiation. These results provided some new insights for our understanding of smoking behaviors in both schizophrenia patients and the general population.

## Methods

### Phenotypes and GWAS datasets

For schizophrenia, we obtained the summary statistics from the PGC GWAS of schizophrenia^1^. This study used 52 independent samples, of them 46 were case control samples of European ancestry, 3 were Asian case control samples and 3 were European family samples. Since the samples were collected from different countries, both the criteria for Diagnostic and Statistical Manual of Mental Disorders (DSM) and International Classification of Diseases (ICD) were used in the diagnosis of the patients. Please see original paper^1^ for details. We selected to use the summary statistics of the 46 European case control samples (32,405 cases and 46,839 controls). For ND-related traits, we used the summary statistics of our cotinine study^65^ and 2 unpublished datasets (manuscripts in preparation). One data used the sum scores of the Fagerström Test for Nicotine Dependence (FTND)^66^ as a trait, which is a commonly used phenotype for ND based on self-reported smoking behaviors. The second data used a single item of the FTND questionnaire, “How soon after you wake up do you smoke your first cigarette”, or time to smoke the first cigarette (TFC) as a trait. This question can be seen as a measure of nicotine withdrawal since the half-life of nicotine in the human body is about 2 hours^67^. Smokers often experience nicotine withdrawal in the morning after not smoking overnight. The third data^65^ used the plasma cotinine concentration (COT) as a trait. Cotinine is the major metabolite of nicotine, and its half-life is much longer than that of nicotine. Therefore, its concentration in plasma can be considered an index of nicotine intake in recent days^68^,^69^. Because the quantity of nicotine intake is one of the most important measures of ND, COT may be considered a measure of ND as well. In these studies, FTND, TFC and COT were treated as quantitative traits. The sample size for FTND was 16,237, excluding the Netherlands Twin Registry sample because some of its subjects were also used in COT GWAS. The sample sizes for TFC and COT were 15,705 and 4,575 respectively. The FTND and TFC measures were derived from the same subjects, therefore, only FTND was used in polygenic analyses. TFC were used only for the identification of shared genes between schizophrenia and ND related phenotypes. The samples used in these 3 ND related GWASs were listed in Supplementary Table S1. All subjects used in this study were of European ancestry.

### Polygenic analyses

Schizophrenia risk scores were calculated for 9 independent smoking related studies (Table S1, n = 10,794) with FTND and CPD measures using the summary statistics from the PGC schizophrenia meta-analysis. The control subjects from the Molecular Genetics of Schizophrenia (MGS) were included in the GWASs of both FTND and PGC schizophrenia, therefore they were excluded from this analysis. Risk scores for COT and FTND were calculated for 13,326 individuals from the NIMH genetics consortium repository (https://www.nimhgenetics.org/). We estimated the risk scores for each trait using the algorithms implemented in the PLINK software^70^. Specifically, the risk score for an individual was the sum of the number of risk alleles multiplied by the logarithm of odds ratio (OR, for schizophrenia) or beta coefficient (for FTND and COT), which was then normalized subsequently by the product of maximal number of risk alleles and log(OR)s/beta coefficients. For each trait, we calculated risk scores at 5 P-value thresholds: 5 × 10^−5^, 5 × 10^−4^, 5 × 10^−3^, 5 × 10^−2^ and 5 × 10^−1^. The numbers of markers used to calculate schizophrenia risk scores at these thresholds were 6,014, 94,804, 268,070, 1,021,476 and 5,370,899. The numbers of markers used for FTND and COT were 731, 6,312, 55,378, 500,542 and 4,752,196; and 1,621, 6,357, 48,575, 473,100, and 4,737,313 respectively. We then tested whether schizophrenia risk scores predicted FTND scores and vice versa using logistic (schizophrenia) and linear regression (FTND scores). Since the number of cigarettes smoked per day (CPD) was available from the FTND datasets, we also tested whether the genetic risk scores for schizophrenia predicted the CPD phenotype. Because we did not have individual genotypes for all datasets used in the COT meta-analyses, we used only the COT risk score to predict schizophrenia diagnosis. Sex, age and study were included as covariates in regression analyses.

### Identification of shared risk genes

While there are papers looking at pleiotropy from a conditional FDR point of view^71^, we arrive to qualitatively similar conclusions using a somewhat simpler approach of family-wise error rate. Our test attempts to discover shared risk genes between schizophrenia and ND using summary statistics from their respective GWASs. To ensure that such a test is not overly influenced by a strong signal in just one trait, we implemented a “weakest link” approach (i.e., choosing the larger P-value of the pair of trait tests at the SNP under investigation)^72^. In more detail, let *X*_*j*_ and *P*_*j*_ be the χ^2^ distributed statistics and their associated (background enrichment adjusted) P-values, *j* = 1, …, *m*, for association tests between the *m* traits and a SNP. As the overlap statistic of all traits we use 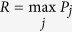 (or, alternatively,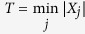). Under the assumption that the trait tests are independent, the P-value (also denoted as overlap P-value) for a given overlap statistic, *r*, at a SNP is 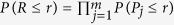. If we further assume that (under the null hypothesis - H_0_) none of the traits is associated with the genetic variant, the overlap P-value simplifies to *P*(*R* ≤ *r*) = *r*^*m*^ (1). Otherwise, *P*(*P*_*j*_ ≤ *r*) can be computed based on the distribution of the j-th trait P-values. For instance, for two phenotype configuration and a putative threshold of 5 × 10^−8^, the parametric version of our method requires that, for a significant pleiotropic signal, the p-values for both phenotypes to be <2.2 × 10^−4^ (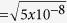). This substantially less than 5 × 10^−8^ p-value threshold is similar in spirit to the one from Andeassen *et al.*^71^ While the overlap p-value (1) does eliminate most of the influence of an extreme signal for one phenotype, it does not eliminate it completely. However, for a putative threshold of 5 × 10^−8^ in (1), under the worst case scenario of an extreme signal in one phenotype, the false positive rate per SNP is still rather small, i.e. 2.2 × 10^−4^. Even more, as seen in Andreassen *et al.*, the false positive rate is likely to be substantially lower. Moreover, a worst-case-scenario 2.2 × 10^−4^ false positive rate is adequate for the pathway analyses^73^. We used FDR^24^ to evaluate the approximate significance of the genetic overlap (described by relation (1)) between schizophrenia and smoking phenotypes. To select promising markers for pathway and network analyses we applied a threshold of q-value ≤ 0.16, corresponding to a factor of 2 in Akaike Information Criterion penalty in a likelihood ratio χ^2^ test with 1 degree of freedom.

### Pathway and network analyses

We conducted pathway enrichment analysis of genes with at least one marker with q-values lower than 0.16 from the joint testing of schizophrenia and COT/FTND/TFC traits. If a marker was within a gene region, it was assigned to the gene; otherwise, it was mapped to its most proximate gene using the 50-kb flanking regions (both 5′ and 3′ sides). Genes identified using SNPs associated with COT, FTND, or TFC were merged for the pathway enrichment analysis, for which we used the hypergeometric test implemented in the tool WebGestalt (2013 update)^74^ and the canonical pathways from the Kyoto Encyclopedia of Genes and Genomes (KEGG) database. We required each pathway to have at least three genes from our gene list and no more than 300 genes from the reference genome. The P-values from hypergeometric tests were further adjusted by the Benjamini-Hochberg method^23^. Only pathways with adjusted P-values < 0.05 were considered statistically significantly enriched.

We further examined pathways interaction using the Characteristic Sub-Pathway Network (CSPN) algorithm^31^,^75^ the human protein-protein interaction (PPI) network^76^. We restricted the analysis specifically to the aforementioned merged gene set and their enriched pathways. In the final step, we selected the significant pathway interaction pairs based on permutation P-values less than 0.05.

## Additional Information

**How to cite this article**: Chen, J. *et al.* Genetic relationship between schizophrenia and nicotine dependence. *Sci. Rep.* **6**, 25671; doi: 10.1038/srep25671 (2016).

## Supplementary Material

Supplementary Information

## Figures and Tables

**Table 1:** Schizophrenia risk score prediction of FTND and CPD.

Threshold (P)	Beta	S.E.	T_value	Pr(>|T|)	R^2^
FTND					
5 × 10^−5^	2.41E-04	0.000269	0.90	0.3708	0
5 × 10^−4^	3.30E-04	0.000235	1.41	0.1599	1.00E-04
**5** × **10**^−**3**^	**3.91E-04**	**0.000194**	**2.01**	**0.0440**	**3.00E-04**
**5** × **10**^−**2**^	**3.53E-04**	**0.000153**	**2.31**	**0.0211**	**4.00E-04**
**5** × **10**^−**1**^	**3.22E-04**	**0.000118**	**2.73**	**0.0063**	**7.00E-04**
CPD					
5 × 10^−5^	1.03E-04	8.77E-05	1.17	0.2424	0
5 × 10^−4^	1.37E-04	7.64E-05	1.79	0.0736	1.00E-04
**5** × **10**^−**3**^	**1.71E-04**	**6.32E-05**	**2.71**	**0.0067**	**5.00E-04**
**5** × **10**^−**2**^	**1.56E-04**	**4.99E-05**	**3.13**	**0.0018**	**6.00E-04**
**5** × **10**^−**1**^	**1.29E-04**	**3.84E-05**	**3.37**	**0.0008**	**8.00E-04**

**Table 2:** Genetic risk score to nicotine dependence prediction of schizophrenia diagnosis*.

Threshold (P)	Beta	S.E.	Wald Z	Pr(>|Z|)	R^2^
Cotinine					
**5** × **10**^−**5**^	**0.0459**	**0.0169**	**2.72**	**0.0066**	**0.0006**
**5** × **10**^−**4**^	**0.0583**	**0.0169**	**3.45**	**0.0006**	**0.0010**
**5** × **10**^−**3**^	**0.0435**	**0.0169**	**2.58**	**0.0100**	**0.0005**
5 × 10^−2^	0.0035	0.0170	0.20	0.8384	3.4E-06
5 × 10^−1^	−0.0226	0.0172	−1.32	0.1886	0.0001
FTND					
5 × 10^−5^	0.0220	0.0242	0.91	0.3641	0.0001
5 × 10^−4^	0.0227	0.0507	0.45	0.6537	2.5E-05
5 × 10^−3^	0.0331	0.0617	0.54	0.5913	3.5E-05
5 × 10^−2^	0.0033	0.0323	0.10	0.9174	1.3E-06
5 × 10^−1^	0.0281	0.0292	0.96	0.3362	0.0001

^*^R^2^ is the Nagelkerke’s R^2^ obtained by the R package fmsb.

**Table 3:** Joint testing of association with schizophrenia and smoking traits.

SNP	Chr	Location (bp)	Z (ND)	Z (SCZ)	P-value	Q-value	Gene symbol	# markers
COT								
rs798015	1	117,320,907	−3.71	−3.69	5.08E-08	0.0020	DA376252	3
rs9850756	3	18,905,739	−3.71	3.62	8.72E-08	0.0030	DA733783	1
rs709071	3	191,426,311	−3.46	3.51	2.85E-07	0.0081	–	1
rs12640124	4	118,775,172	3.61	−3.50	2.12E-07	0.0063	BX089737	3
rs2442720	6	31,320,277	−3.49	4.62	2.26E-07	0.0067	HLA-B	1
rs11779524	8	8,618,613	3.85	3.81	1.89E-08	0.0008	CF594265	4
rs56235824	9	11,039,320	3.44	3.55	3.38E-07	0.0090	–	1
rs11788261	9	138,611,139	3.80	3.51	1.95E-07	0.0060	KCNT1	2
rs8042374	15	78,908,032	7.69	7.04	3.48E-24	1.68E-17	CHRNA5/CHRNA3/CHRNB4	270
rs6650723	18	53,524,269	3.76	3.51	2.07E-07	0.0062	DA696352/LOC101927273	5
rs7228837	18	75,817,606	4.23	3.65	6.64E-08	0.0024	LINC01029	19
FTND								
rs56335113	1	30,427,639	3.80	5.85	2.02E-08	0.0021	–	10
rs36025078	2	155,883,716	−3.90	−3.59	1.13E-07	0.0061	–	1
rs188499496	3	38,450,183	−3.93	−3.62	8.82E-08	0.0050	XYLB	2
rs11917643	3	64,171,754	3.62	−4.17	8.41E-08	0.0048	PRICKLE2	6
rs76923559	4	34,053,926	−3.93	−3.79	2.35E-08	0.0023	LOC101928622	3
rs147093127	5	152,086,293	3.67	4.01	5.70E-08	0.0037	LINC01470	1
rs9322751	6	104,015,759	−3.51	−3.54	2.03E-07	0.0089	HY157071	6
rs4994764	7	1,928,662	−3.90	−4.32	9.17E-09	0.0012	MAD1L1	18
rs3910267	11	130,810,282	−3.84	5.71	1.50E-08	0.0018	SNX19	21
rs147144681	15	78,900,908	−7.62	−5.55	7.85E-16	5.66E-09	CHRNA5/CHRNA3/CHRNB4	106
TFC								
rs4658015	1	196,053,435	3.51	3.36	5.91E-07	0.0094	–	1
rs1069267	3	38,435,023	−3.63	−4.43	8.02E-08	0.0015	XYLB	3
rs11722779	4	103,827,488	4.22	−4.30	6.00E-10	0.0001	MANBA/UBE2D3/SLC9B2	315
rs2717737	8	18,459,343	−3.36	3.73	6.05E-07	0.0096	PSD3	1
rs13264022	8	21,293,046	3.50	4.01	2.16E-07	0.0038	DA902558	15
rs7959287	12	103,601,638	3.84	4.49	1.54E-08	0.0003	–	5
rs9594516	13	41,849,360	3.64	3.77	7.33E-08	0.0014	MTRF1	1
rs112531467	13	43,865,047	−3.66	3.52	1.86E-07	0.0033	ENOX1	1
rs181676509	14	104,242,531	−3.67	−3.98	5.73E-08	0.0011	C14ORF2	15
rs147144681	15	78,900,908	−5.50	−5.55	1.49E-15	1.08E-08	CHRNA5/CHRNA3/CHRNB4	72
rs17665477	16	6,700,889	−3.69	3.80	5.16E-08	0.0010	RBFOX1	11
rs12462853	19	5,538,936	3.45	−3.39	4.82E-07	0.0078	TINCR	2
rs62202174	20	20,461,125	−3.38	−4.24	5.26E-07	0.0085	RALGAPA2	1
rs5758274	22	41,664,539	3.64	−4.45	7.43E-08	0.0014	RANGAP1	1
rs8135804	22	42,334,660	3.40	3.57	4.54E-07	0.0074	CENPM	12

**Table 4:** Pathways enriched in schizophrenia and smoking traits.

Pathway	Genes found in pathway	# gene in pathway	# gene observed	# gene expected	Observed/expected ratio	Raw P-value	Adjusted P-value
Calcium signaling pathway	ATP2B2, CACNA1C, CACNA1I, CHRM3, ITPR1, ITPR2	177	6	0.57	10.59	2.41E-05	0.0004
Long-term potentiation	CACNA1C, ITPR1, ITPR2, PPP1CB	70	4	0.22	17.86	7.81E-05	0.0007
Neuroactive ligand-receptor interaction	CHRM3, CHRNA3, CHRNA5, CHRNB4, NR3C1, THRB	272	6	0.87	6.89	0.0003	0.0011
Salivary secretion	ATP2B2, CHRM3, ITPR1, ITPR2	89	4	0.28	14.05	0.0002	0.0011
Pancreatic secretion	ATP2B2, CHRM3, ITPR1, ITPR2	101	4	0.32	12.38	0.0003	0.0011
Vascular smooth muscle contraction	CACNA1C, ITPR1, ITPR2, PPP1CB	116	4	0.37	10.78	0.0005	0.0013
Oocyte meiosis	ITPR1, ITPR2, PPP1CB, SPDYA	112	4	0.36	11.16	0.0005	0.0013
Phosphatidylinositol signaling system	INPP5K, ITPR1, ITPR2	78	3	0.25	12.02	0.0020	0.0036
Gastric acid secretion	CHRM3, ITPR1, ITPR2	74	3	0.24	12.67	0.0018	0.0036
Arrhythmogenic right ventricular cardiomyopathy (ARVC)	CACNA1C, ITGAV, SGCD	74	3	0.24	12.67	0.0018	0.0036
Hypertrophic cardiomyopathy (HCM)	CACNA1C, ITGAV, SGCD	83	3	0.27	11.30	0.0024	0.0039
Dilated cardiomyopathy	CACNA1C, ITGAV, SGCD	90	3	0.29	10.42	0.0031	0.0046
GnRH signaling pathway	CACNA1C, ITPR1, ITPR2	101	3	0.32	9.28	0.0042	0.0058
Cell adhesion molecules (CAMs)	ITGAV, NCAM2, PTPRM	133	3	0.43	7.05	0.0091	0.0117
Alzheimer’s disease	CACNA1C, ITPR1, ITPR2	167	3	0.53	5.61	0.0167	0.0188
Regulation of actin cytoskeleton	CHRM3, ITGAV, PPP1CB	213	3	0.68	4.40	0.0313	0.0331
