# Supplementary material for: Genetic Relationship between Schizophrenia and Nicotine Dependence
Source: Sci Rep. 2016 May 10;6:25671. doi: 10.1038/srep25671 (PMC4862382; doi:10.1038/srep25671)
Supplement: Supplementary Information [file srep25671-s1.pdf]

Genetic Relationship between Schizophrenia and Nicotine Dependence

Jingchun Chen[1,], Silviu-Alin Bacanu[2,*], Hui Yu[3], Zhongming Zhao[3], Peilin Jia[3], Kenneth S Kendler[2], Henry R. Kranzler[4], Joel Gelernter[5], Lindsay Farrer[6], Camelia Minica[7], Rene Pool[7], Yuri Milaneschi[8], Dorret I Boomsma[7], Brenda W. J. H. Penninx[7], Rachel F. Tyndale[9], Jennifer J. Ware[10],Cotinine meta-analysis group, FTND meta-analysis group, Jacqueline Vink[7], Jaakko Kaprio [11,12,13], Marcus Munafò[10,14], Xiangning Chen[1, #]*

Supplementary Table S1. Data sets used in FTND and cotinine genome-wide meta-analyses

Samples	N (phenotype)	Phenotype	SCZ risk score	Reference
ALSPAC	258/161/193	COTININE/FTND/TFC	No	[1]
CARDIA	387	COTININE	No	[2]
FinnTwin	145	COTININE	No	[3-5]
FINRISK	218	COTININE	No	[6, 32]
Framingham	93	COTININE	No	[7]
GenMets	485	COTININE	No	[8]
MESA	189	COTININE	No	[9]
NESDA	808	COTININE	No	[10]
NTR	897/2436/2484	COTININE/FTND/TFC	No	[11-16]
TwinsUK	676	COTININE	No	[17]
YFS	392	COTININE	No	[18]
SAGE	2042/2042	FTND/TFC	Yes	[19-21]
SC	2479/2479	FTND/TFC	Yes	[22,23]
MGS	2496/2496	FTND/TFC	No	[24]
COPD	320/320	FTND/TFC	Yes	[25]
ISIB	299/265	FTND/TFC	Yes	[26]
CIDR370v1	166/574	FTND/TFC	Yes	[27]
CIDR370v3	251/598	FTND/TFC	Yes	[27]
CIDRc3	1710	TFC	Yes	[28]
CIDRc4	477	TFC	Yes	[29]
EAGLE	3051	FTND	Yes	[30]
AUTW	190/190	FTND/TFC	No	-
CEDAR	294/294	FTND/TFC	No	-
FIN1	1563/1573	FTND/TFC	No	[3-5]
FIN2	811/811	FTND/TFC	No	[3-5]
Yale_Penn	2114/2117	FTND/TFC	No	[31]

References

[1] Boyd A, Golding J, Macleod J, Lawlor DA, Fraser A, Henderson J et al. Cohort Profile: the 'children of the 90s'--the index offspring of the Avon Longitudinal Study of Parents and Children. Int J Epidemiol 2013, 42: 111-127.

[2] Hughes GH, Cutter G, Donahue R, Friedman GD, Hulley S, Hunkeler E et al. Recruitment in the Coronary Artery Disease Risk Development in Young Adults (Cardia) Study. Control Clin Trials 1987, 8: 68S-73S.

[3] Kaprio J. Twin studies in Finland 2006. Twin Res Hum Genet 2006, 9: 772-777.

[4] Kaprio J. The Finnish Twin Cohort Study: an update. Twin Res Hum Genet 2013, 16: 157-162.

[5] Loukola A, Wedenoja J, Keskitalo-Vuokko K, Broms U, Korhonen T, Ripatti S et al. Genome-wide association study on detailed profiles of smoking behavior and nicotine dependence in a twin sample. Mol Psychiatry 2014, 19: 615-624.

[6] Vartiainen E, Seppala T, Lillsunde P, Puska P. Validation of self reported smoking by serum cotinine measurement in a community-based study. J Epidemiol Community Health 2002, 56: 167-170.

[7] Wang TJ, Larson MG, Vasan RS, Cheng S, Rhee EP, McCabe E et al. Metabolite profiles and the risk of developing diabetes. Nat Med 2011, 17: 448-453.

[8] Keskitalo K, Broms U, Heliovaara M, Ripatti S, Surakka I, Perola M et al. Association of serum cotinine level with a cluster of three nicotinic acetylcholine receptor genes (CHRNA3/CHRNA5/CHRNB4) on chromosome 15. Hum Mol Genet 2009, 18: 4007-4012.
[9] Bild DE, Bluemke DA, Burke GL, Detrano R, Diez Roux AV, Folsom AR et al. Multi-ethnic study of atherosclerosis: objectives and design. Am J Epidemiol 2002, 156: 871-881.
[10] Penninx BW, Beekman AT, Smit JH, Zitman FG, Nolen WA, Spinhoven P et al. The Netherlands Study of Depression and Anxiety (NESDA): rationale, objectives and methods. Int J Methods Psychiatr Res 2008, 17: 121-140.
[11] Boomsma DI, Vink JM, van Beijsterveldt TC, de Geus EJ, Beem AL, Mulder EJ et al. Netherlands Twin Register: a focus on longitudinal research. Twin Res 2002, 5: 401-406.
[12] Boomsma DI, de Geus EJ, Vink JM, Stubbe JH, Distel MA, Hottenga JJ et al. Netherlands Twin Register: from twins to twin families. Twin Res Hum Genet 2006, 9: 849-857.
[13] Willemsen G, de Geus EJ, Bartels M, van Beijsterveldt CE, Brooks AI, Estourgie-van Burk GF et al. The Netherlands Twin Register biobank: a resource for genetic epidemiological studies. Twin Res Hum Genet 2010, 13: 231-245.
[14] Willemsen G, Vink JM, Abdellaoui A, den BA, van Beek JH, Draisma HH et al. The Adult Netherlands Twin Register: twenty-five years of survey and biological data collection. Twin Res Hum Genet 2013, 16: 271-281.
[15] Scheet P, Ehli EA, Xiao X, van Beijsterveldt CE, Abdellaoui A, Althoff RR et al. Twins, tissue, and time: an assessment of SNPs and CNVs. Twin Res Hum Genet 2012, 15: 737-745.
[16] Vink JM, Willemsen G, Beem AL, Boomsma DI. The Fagerstrom Test for Nicotine Dependence in a Dutch sample of daily smokers and ex-smokers. Addict Behav 2005, 30: 575-579.
[17] Moayyeri A, Hammond CJ, Valdes AM, Spector TD. Cohort Profile: TwinsUK and healthy ageing twin study. Int J Epidemiol 2013, 42: 76-85.
[18] Raitakari OT, Juonala M, Ronnemaa T, Keltikangas-Jarvinen L, Rasanen L, Pietikainen M et al. Cohort profile: the cardiovascular risk in Young Finns Study. Int J Epidemiol 2008, 37: 1220-1226.
[19] Saccone SF, Hinrichs AL, Saccone NL, Chase GA, Konvicka K, Madden PA et al. Cholinergic nicotinic receptor genes implicated in a nicotine dependence association study targeting 348 candidate genes with 3713 SNPs. Hum Mol Genet 2007, 16: 36-49.
[20] Bierut LJ, Saccone NL, Rice JP, Goate A, Foroud T, Edenberg H et al. Defining alcohol-related phenotypes in humans. The Collaborative Study on the Genetics of Alcoholism. Alcohol Res Health 2002, 26: 208-213.
[21] Bierut LJ, Strickland JR, Thompson JR, Afful SE, Cottler LB, Shi J et al. Drug use and dependence in cocaine dependent subjects, community-based individuals, and their siblings. Drug Alcohol Depend 2008, 95: 14-22.
[22] Piper ME, Smith SS, Schlam TR, Fiore MC, Jorenby DE, Fraser D et al. A randomized placebo-controlled clinical trial of 5 smoking cessation pharmacotherapies. Arch Gen Psychiatry 2009, 66: 1253-1262.
[23] Japuntich SJ, Leventhal AM, Piper ME, Bolt DM, Roberts LJ, Fiore MC et al. Smoker characteristics and smoking-cessation milestones. Am J Prev Med 2011, 40: 286-294.
[24] Shi J, Levinson DF, Duan J, Sanders AR, Zheng Y, Pe'er I et al. Common variants on chromosome 6p22.1 are associated with schizophrenia. Nature 2009, 460: 753-757.
[25] Regan EA, Hokanson JE, Murphy JR, Make B, Lynch DA, Beaty TH et al. Genetic epidemiology of COPD (COPDGene) study design. COPD 2010, 7: 32-43.
[26] Prescott CA, Sullivan PF, Kuo PH, Webb BT, Vittum J, Patterson DG et al. Genomewide linkage study in the Irish affected sib pair study of alcohol dependence: evidence for a susceptibility region for symptoms of alcohol dependence on chromosome 4. Mol Psychiatry 2006, 11: 603-611.
[27] Whitfield JB, Zhu G, Madden PA, Neale MC, Heath AC, Martin NG. The genetics of alcohol intake and of alcohol dependence. Alcohol Clin Exp Res 2004, 28: 1153-1160.

[28] Shand FL, Degenhardt L, Nelson EC, Mattick RP, Malison RT, Kalayasiri R et al. Predictors of social anxiety in an opioid dependent sample and a control sample Inter-rater reliability and concurrent validity of DSM-IV opioid dependence in a Hmong isolate using the Thai version of the Semi-Structured Assessment for Drug Dependence and Alcoholism (SSADDA). J Anxiety Disord 2010, 24: 49-54.

[29] Sherva R, Kranzler HR, Yu Y, Logue MW, Poling J, Arias AJ et al. Variation in nicotinic acetylcholine receptor genes is associated with multiple substance dependence phenotypes. Neuropsychopharmacology 2010, 35: 1921-1931.

[30] Landi MT, Consonni D, Rotunno M, Bergen AW, Goldstein AM, Lubin JH et al. Environment And Genetics in Lung cancer Etiology (EAGLE) study: an integrative population-based case-control study of lung cancer. BMC Public Health 2008, 8: 203-

[31] Han S, Yang BZ, Kranzler HR, Oslin D, Anton R, Gelernter J. Association of CHRNA4 polymorphisms with smoking behavior in two populations. Am J Med Genet B Neuropsychiatr Genet 2011, 156B: 421-429.

[32] Broms U, Pennanen M, Patja K, Ollila H, Korhonen T, Kankaanpää A, Haukkala A, Tuulio-Henriksson A, Koskenvuo M, Kronholm E, Laatikainen T, Peltonen M, Partonen, T, Kaprio J. Diurnal Evening Type is Associated with Current Smoking, Nicotine Dependence and Nicotine Intake in the Population Based National FINRISK 2007 Study. J Addict Res Ther. 2012 Jan 25;S2. pii: 002. PubMed PMID: 22905332; PubMed Central PMCID: PMC3422076.

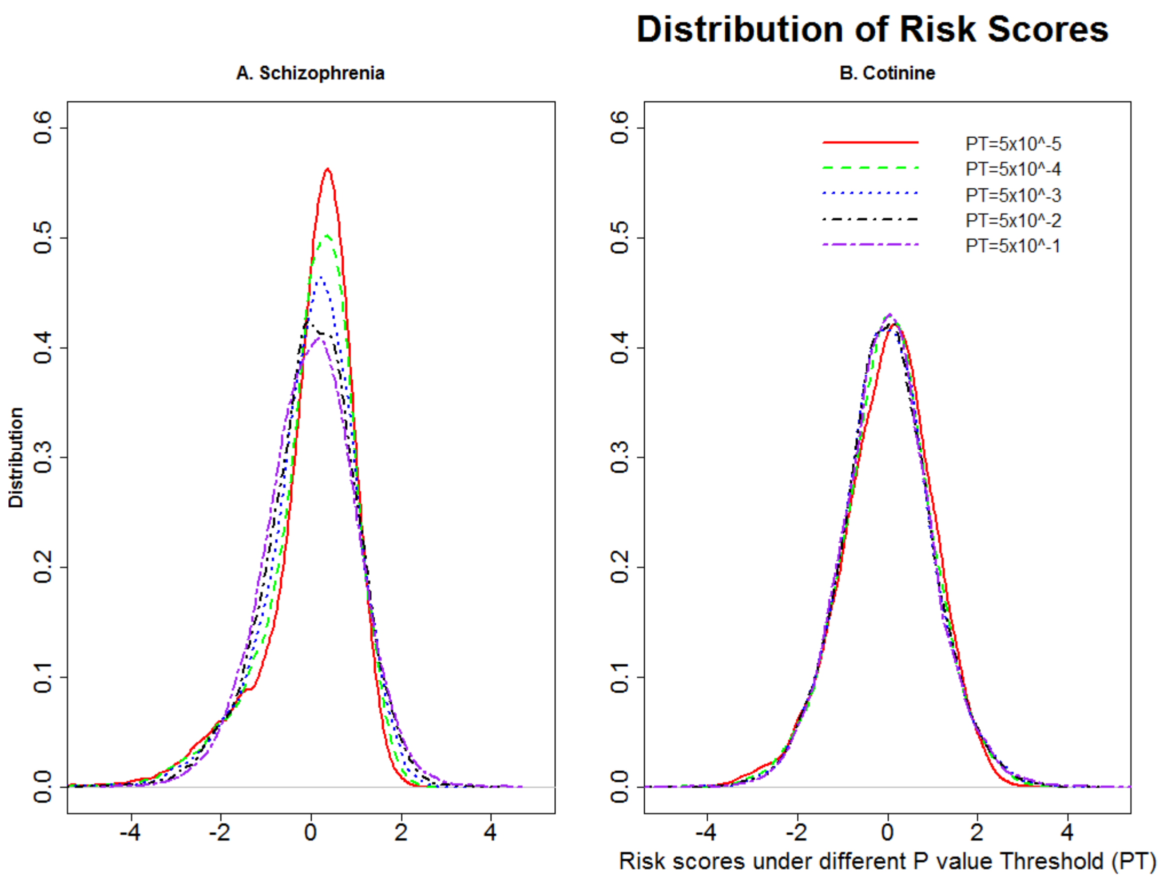

Supplementary Figure S1. Distributions of genetic risk scores used in this study.
